# The cost-effectiveness analysis of serplulimab versus regorafenib for treating previously treated unresectable or metastatic microsatellite instability-high or deficient mismatch repair colorectal cancer in China

**DOI:** 10.3389/fonc.2023.1113346

**Published:** 2023-04-26

**Authors:** Yue Ma, Jiting Zhou, Yuxin Ye, Xintian Wang, Aixia Ma, Hongchao Li

**Affiliations:** ^1^ School of International Pharmaceutical Business, China Pharmaceutical University, Nanjing, China; ^2^ Center for Pharmacoeconomics and Outcomes Research, China Pharmaceutical University, Nanjing, China

**Keywords:** cost-effectiveness analysis, MSI-H/dMMR, colorectal cancer, serplulimab, regorafenib, China

## Abstract

**Objective:**

The aim of this study was to investigate the cost-effectiveness of serplulimab versus regorafenib in previously treated unresectable or metastatic microsatellite instability-high (MSI-H)/deficient mismatch repair (dMMR) colorectal cancer in China.

**Methods:**

From the perspective of China’s health-care system, a Markov model with three health states (progression free, progression, death) was developed for estimating the costs and health outcomes of serplulimab and regorafenib. Data for unanchored matching-adjusted indirect comparison (MAIC), standard parametric survival analysis, the mixed cure model, and transition probabilities calculation were obtained from clinical trials (ASTRUM-010 and CONCUR). Health-care resource utilization and costs were derived from government-published data and expert interviews. Utilities used to calculate quality-adjusted life years (QALYs) were obtained from clinical trials and literature reviews. The primary outcome was the incremental cost-effectiveness ratio (ICER) expressed as cost/QALY gained. Four scenarios were considered in scenario analysis: (a) using original survival data without conducting MAIC; (b) limiting the time horizon to the follow-up time of the clinical trial of serplulimab; (c) adopting a fourfold increase in the risk of death; and (d) applying utilities from two other sources. One-way sensitivity analysis and probabilistic sensitivity analysis were also performed to assess the uncertainty of the results.

**Results:**

In the base-case analysis, serplulimab provided 6.00 QALYs at a cost of $68,722, whereas regorafenib provided 0.69 QALYs at a cost of $40,106. Compared with that for treatment with regorafenib, the ICER for treatment with serplulimab was $5,386/QALY, which was significantly lower than the triple GDP per capita of China in 2021 ($30,036), which was the threshold used to define the cost-effectiveness. In the scenario analysis, the ICERs were $6,369/QALY, $20,613/QALY, $6,037/QALY, $4,783/QALY, and $6,167/QALY, respectively. In the probabilistic sensitivity analysis, the probability of serplulimab being cost-effective was 100% at the threshold of $30,036/QALY.

**Conclusion:**

Compared with regorafenib, serplulimab is a cost-effective treatment for patients with previously treated unresectable or metastatic MSI-H/dMMR colorectal cancer in China.

## 1 Introduction

Colorectal cancer (CRC) is the third most commonly diagnosed cancer and the second leading cause of cancer-related death worldwide (1). The incidence and mortality of CRC have been rising in China, with more than 408,000 newly diagnosed cases and 195,600 deaths occurring in 2016 (2), and age have no effect on survival outcomes (3). Microsatellite instability-high (MSI-H)/deficient mismatch repair (dMMR) is a subtype of CRC that accounts for approximately 4%–5% of patients with advanced CRC (4), immunohistochemistry and/or MSI test are recommended in colon cancer screening for defective DNA mismatch repair (5).

Previous studies have indicated that patients with MSI-H/dMMR CRC are less responsive to chemotherapy than patients with microsatellite stable/proficient mismatch repair CRC, but show higher sensitivity to immune checkpoint inhibitors (ICIs) (6–8). In recent years, four ICIs, including three programmed death receptor-1 (PD-1) inhibitors (serplulimab, pembrolizumab, and tislelizumab) and one programmed death-ligand 1 (PD-L1) inhibitor (envafolimab), for MSI-H/dMMR CRC have obtained regulatory approval in China. The Guidelines of the Chinese Society of Clinical Oncology (CSCO) for Immune Checkpoint Inhibitor Clinical Practice 2022 recommended that patients with MSI-H/dMMR CRC who have progressed following chemotherapy should accept ICIs for subsequent treatment (9). However, to date, none of these ICIs has been added to China’s national reimbursement drug list (NRDL), imposing a heavy financial burden on patients. Thus, there is an urgent need to generate evidence for decision-making regarding which of these ICIs of MSI-H/dMMR CRC should be added to the NRDL.

Although the clinical trials that assessed the efficacy and safety of the four abovementioned ICIs all showed great survival benefits (the median overall survival (OS) of all four drugs were either not reached or not reported) (10–12), after comparing their characteristics, we chose serplulimab for the target intervention in our study. Our rationale is as follows: (1) Serplulimab showed great clinical efficacy for previously treated MSI-H/dMMR CRC patients with a median PFS of 9.83 months; (2) With more locally developed ICIs appearing on the Chinese market, price competition continues to intensify, resulting in lower prices for locally produced ICIs than for pembrolizumab. As one of the locally produced ICIs, serplulimab offers an affordable price and greater accessibility; (3) All of the patients recruited for the clinical trial of serplulimab (ASTRUM-010) were Chinese, which provides better support for Chinese decision-making; and (4) Individual patient data (IPD) were available for serplulimab treatment. Serplulimab is a fully humanized monoclonal antibody against PD-1 and the first locally produced drug for which an application has been lodged for listing in China in relation to treatment of MSI-H/dMMR solid tumors. Thus, it is worthwhile evaluating the cost-effectiveness of the use of serplulimab for the treatment of MSI-H/dMMR CRC.

In China, innovative drugs need to be compared with currently listed drugs being included in the NRDL, based on the requirements of China’s national health-care security administration (NHSA) (13). Regorafenib is a small-molecule multikinase inhibitor that targets signaling pathways implicated in tumor angiogenesis, oncogenesis, and the tumor microenvironment (14). In 2018, regorafenib was approved as a third-line treatment for metastatic CRC and added to the NRDL based on the results of the CONCUR trial (14), in which more than 80% of the patients were Chinese. Experts have confirmed that regorafenib is also widely used for previously treated MSI-H/dMMR CRC in clinical practice when there is a lack of ICIs. Thus, regorafenib was selected for comparison with serplulimab. The clinical trial data showed that the median OS (8.8 months vs. 6.3 months, hazard ratio [HR]: 0.55, 95% confidence interval [CI]: 0.40–0.77) and the median PFS (3.2 months vs. 1.7 months, HR: 0.31, 95% CI: 0.22–0.44) were significantly prolonged by using regorafenib versus a placebo for advanced CRC (14).

Cost-effectiveness analysis (CEA) is an analytic method that is used for quantifying the relative health benefits and costs of two or more alternative interventions within a consistent framework, which helps health-care decision-makers to choose the optimal intervention when resources are limited, and has been used extensively in many countries including the US, England, Canada, and Australia (15, 16). To our knowledge, few CEAs have been published regarding the treatment of MSI-H/dMMR CRC. Thus, in this study, we investigate the cost-effectiveness of serplulimab versus regorafenib for previously treated unresectable or metastatic MSI-H/dMMR CRC in China from the health-care system perspective and provide evidence for decision-making regarding adjustments to the NRDL.

## 2 Methods

### 2.1 Analytical overview and model structure

#### 2.1.1 Target population

The target population was adult patients with previously treated unresectable or metastatic MSI-H/dMMR CRC. This is consistent with the patient population in the ASTRUM-010 trial.

#### 2.1.2 Target intervention and comparators

In our analysis, treatments with serplulimab and regorafenib were compared. In the case of serplulimab, the recommended dose is 3 mg/kg administered intravenously every two weeks for up to two years. Regarding regorafenib, the recommended dose is 160 mg daily for the first 21 days of each 28-day cycle until either disease progression or death.

#### 2.1.3 Model structure

**Figure 1:**
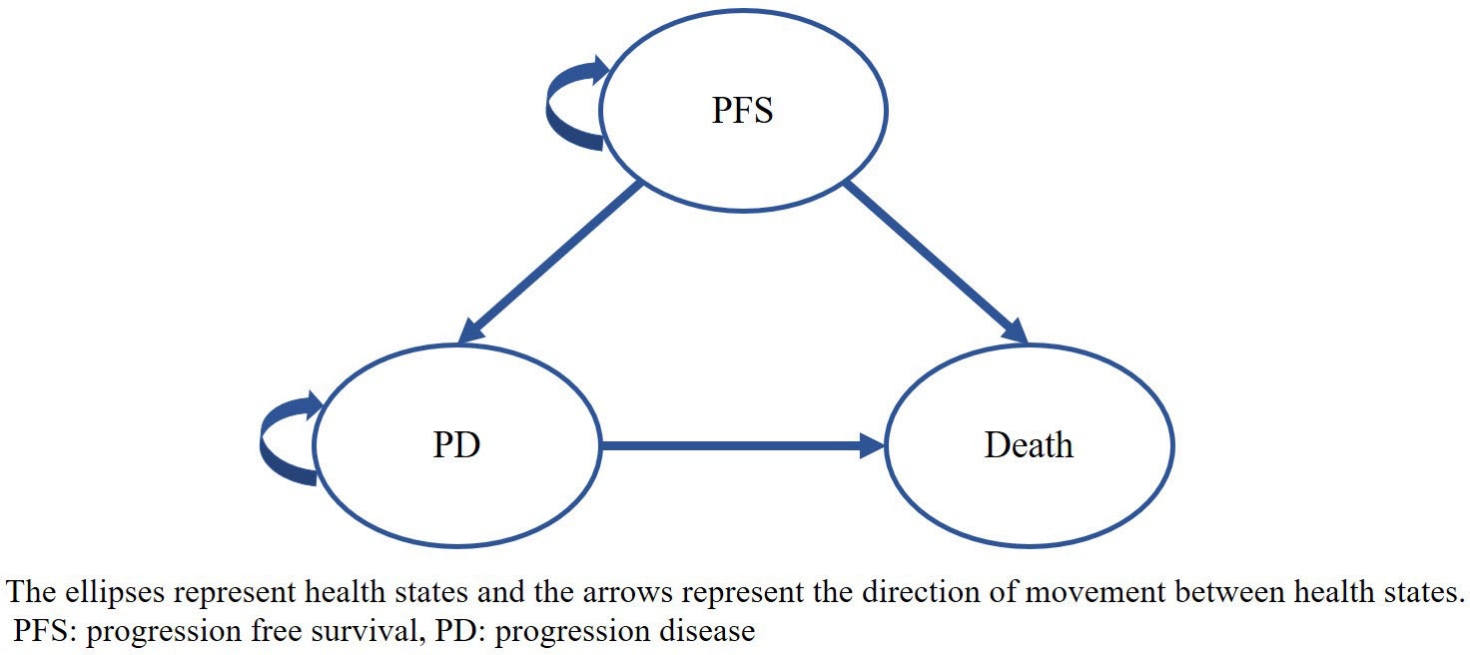
Markov model structure The ellipses represent health states and the arrows represent the direction of movement between health states. PFS: progression free survival, PD, progression disease.

A three-state transition Markov model (see **Figure 1**) was developed using Microsoft Excel 2019, including PFS, progression of disease (PD) and death, which reflected the natural disease history and had been routinely adopted in previous studies on the treatment of CRC (17). In this model, we assumed that patients started in the PFS state, in which they were treated with either serplulimab or regorafenib, and grade 3 or above adverse events (AEs) occurring in the PFS state were also considered. At the end of each cycle, patients whose disease had progressed, transferring to the PD state, received subsequent treatment. Death was treated as an absorbing state that could occur in either the PFS or PD states. The transition probabilities between health states were estimated based on survival curves.

Each model cycle was set at four weeks to accommodate the differing administration regimes for serplulimab and regorafenib as outlined above. A lifetime horizon was used to capture long-term outcomes until more than 99% of patients died in both treatment groups. The primary outcomes of our model included the total cost, life-years (LYs), quality-adjusted life-years (QALYs), and the incremental cost-effectiveness ratio (ICER). An annual discount rate of 5% was applied to both costs and effectiveness, in line with China’s Guidelines for Pharmacoeconomic Evaluations 2020 (18). The willingness-to-pay (WTP) threshold of triple GPD per capita ($36,036 per QALY gained) was used to determine the cost-effectiveness of treatment, also as recommended by China’s Guidelines for Pharmacoeconomic Evaluations 2020 (18, 19).

### 2.2 Model inputs

#### 2.2.1 Clinical efficacy

The clinical efficacy of serplulimab was derived from the ASTRUM-010 trial, which was used to evaluate efficacy and safety in patients with MSI-H/dMMR solid tumors who had either progressed on or been intolerant to standard therapies, and thus we screened the IPD for MSI-H/dMMR CRC. The clinical efficacy of regorafenib was derived from aggregated data from the CONCUR trial, and the IPD were reconstructed using the method developed by Guyot et al. (20).

Because of an absence of direct comparisons between serplulimab and regorafenib and the presence of baseline differences between the two treatments, the relative efficacy of serplulimab compared with that of regorafenib was estimated using the unanchored matching-adjusted indirect comparison (MAIC) method. This statistical method can reduce cross-trial differences in patients’ baseline characteristics and provide comparative evidence with less bias (21). Using the IPD from the ASTRUM-010 trial, the MAIC was performed using prognostic factors and treatment effect modifiers (age, gender, Eastern Cooperative Oncology Group (ECOG) performance status, histology, metastatic site, previous systemic anticancer treatment lines, and previous targeted biological treatment). The original and reweighted baseline characteristics of the patients in the two trials are shown in **Appendix Table 1**. The corresponding adjusted Kaplan–Meier (KM) curves are shown in **Appendix Figure 1**.

**Figure 2:**
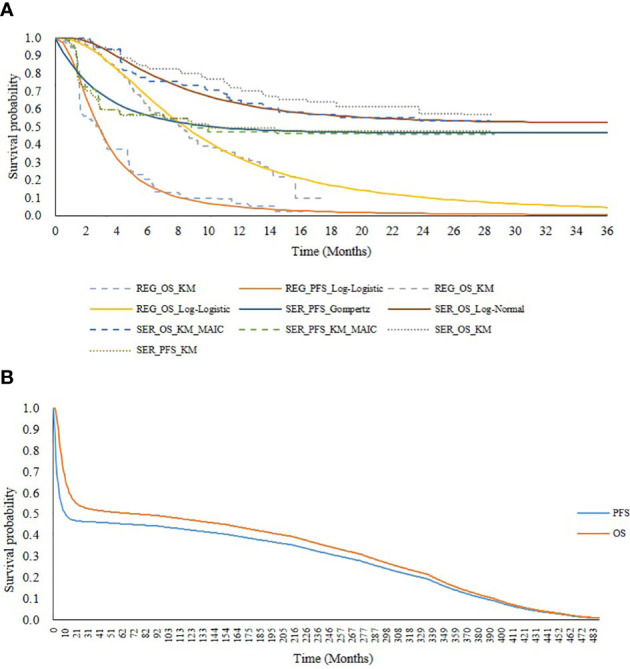
Survival curves for serplulimab and regorafenib. **(A)** presents extrapolation and fitting of survival outcomes from the ASTRUM-010 and CONCUR trial. **(B)** presents PFS and OS curves adjusted with increase of death risk in serplulimab overtime horizon. SER, serplulimab; REG, regorafenib; KM, Kaplan-Meier; PFS, progression free survival; OS, overall survival; MAIC, matching-adjusted indirect comparison.

Using the adjusted IPD for serplulimab and the reconstructed IPD for regorafenib, we extrapolated the survival outcomes beyond the follow-up period. Regarding the OS curve of serplulimab, the median OS was not reached, presenting an immature L-shaped platform, indicating that some patients were long-term survivors. In an effort to reduce bias in the OS estimates, a mixture cure model was adopted. Regarding the other curves, including the PFS curve for serplulimab and the OS curve and PFS curve for regorafenib, six standard parametric models (exponential, gamma, Weibull, log-normal, log-logistic, and Gompertz distributions) were fitted and then extrapolated over the lifetime horizon. Best-fit curves were selected based on the Akaike information criterion (AIC), Bayesian information criterion (BIC), and visual inspection. The AIC and BIC for all fitted curves are shown in **Appendix Table 2** and **Appendix Table 3**. Finally, for serplulimab, the Gompertz distribution was selected to fit the PFS curve and the log-normal distribution in the mixture cure model was selected to fit the OS curve, while for regorafenib, the log-logistic distribution was selected to fit both the PFS and OS curves (see **Figure 2A**).

To address the plateau problem of the survival curve for serplulimab and reduce the uncertainty of our long-term extrapolation, we increased the extra risk of death in this model. Generally, the risk of death of cured patients (patients in the plateau of the survival curve) is assumed to be the same as that for the survival curve (22). However, because patients who are considered to be clinically cured actually have a higher risk of death than the general population, we increased the risk of death for cured patients. The risk of death for patients in both the PFS and PD states was assumed to be twice that of the general population in the base-case analysis based on the NICE guidance (23) and expert opinion. Background mortality rates for various age groups were obtained from China’s Population & Employment Statistical Yearbook 2020 (24). In addition, to ensure the consistency of assumptions between the two treatments, the increased risk of death was also applied to the regorafenib survival curves. The PFS and OS curves adjusted for the increased risk of death are shown in **Figure 2B**.

#### 2.2.2 Resource use and costs

From the health-care system perspective, our analysis only included direct medical costs, including the costs of drug acquisition, diagnosis, patient monitoring, AE management, hospitalization, subsequent treatment, and end-of-life care. These costs are summarized in **Table 1**. All costs were converted into 2022 US dollars at an exchange rate of 6.7413 yuan = one US dollar (31).

**Table 1:** Key parameters in the model.

Parameter	Serplulimab	Regorafenib	Reference
Cost ($)			
Diagnosis	519.26	519.26	Expert opinion and medical service price list^*^
Drug acquisition costs per cycle	3,315.68	2,149.19	(25)
Patient monitoring costs per cycle			(9) and medical service price list^*^
The first 3 years in PFS state	75.65	113.48	
The 4^th^-5^th^ year in PFS state	37.83	37.83	
After 5^th^ year in PFS state	18.91	18.91	
In PD state	75.65	113.48	
AE management			Expert opinion and (25)
Anemia	6.96	–	
Hyperbilirubinemia	76.16	37.92	
Impaired liver function	76.16	–	
Alanine aminotransferase elevated	76.16	37.92	
Aspartate aminotransferase elevated	–	37.92	
Lung infection	88.85	–	
Neutropenia	62.62	84.93	
Leukopenia	62.62	26.48	
Thrombocytopenia	–	2,139.77	
Diarrhea	2.95	12.04	
Myalgia	3.28	–	
Hypertension	0.18	0.18	
Hand foot skin reaction	–	16.18	
Maculopapular rash	–	17.63	
Subsequent treatment costs per cycle	749.71	1,799.71	Expert opinion and (25)
Hospitalization costs	0.00	0.00	Expert opinion and medical service price list^*^
In PFS state	73.38	0.00	
In PD state	110.08	146.77	
Administration costs per cycle	0.00	0.00	Expert opinion and medical service price list^*^
In PFS state	2.97	0.00	
In PD state	1.67	1.80	
End-of-life care costs	2,046.84	2,046.84	(26)
Utility values			
PFS	0.84	0.84	(14)
PD	0.57	0.57	(14)
Disutility values			
Anemia	0.085	–	(27)
Hyperbilirubinemia	0	0	/
Impaired liver function	0	–	/
Alanine aminotransferase elevated	0	0	/
Aspartate aminotransferase elevated	–	0	/
Lung infection	0.195	–	(28)
Neutropenia	0.0607	0.0607	(27)
Leukopenia	0.0607	0.0607	(27)
Thrombocytopenia	–	0.19	(29)
Diarrhea	0.07	0.07	(29)
Creatine kinase elevated	0	–	/
Hypertension	0.04	0.04	(29)
Hand foot skin reaction	–	0.116	(30)
Maculopapular rash	–	0.03248	(27)
**Incidence of AEs (grade 3 or above)**			ASTRUM-010 and (14)
Anemia	10.81%	–	
Hyperbilirubinemia	6.76%	6.00%	
Impaired liver function	5.41%	–	
Alanine aminotransferase elevated	1.35%	7.00%	
Aspartate aminotransferase elevated	–	6.00%	
Lung infection	2.70%	–	
Neutropenia	4.05%	2.00%	
Leukopenia	2.70%	2.00%	
Thrombocytopenia	–	3.00%	
Diarrhea	2.70%	1.00%	
Creatine kinase elevated	2.70%	–	
Hypertension	2.70%	11.00%	
Hand foot skin reaction	–	16.00%	
Maculopapular rash	–	4.00%	

*The prices were median values form medical service price list of 10 representative provinces or cities in China.

- represents that no such adverse reactions occurred in the corresponding group. PFS, Progression free survival; PD, Progression disease; AE, Adverse event.

Drug acquisition costs. Drug acquisition costs included the cost of the drugs and administration costs. Drug costs were calculated based on dosage and treatment duration, as discussed above. The price of serplulimab was $828.92 per 100 mg and the price of regorafenib was $102.34 per 160 mg based on the MENET database (25). To simplify the calculation of the cost of serplulimab, it was assumed that the mean weight of patients was 65 kg, in accordance with NHSA requirements (13). Administration costs, which referred to the cost of intravenous infusion in our study, were included for serplulimab patients. The cost of each intravenous infusion was $1.48 based on the median value of the price of medical services in 10 representative Chinese provinces or cities. Because regorafenib is an orally administered drug, no administration costs were included for regorafenib patients.

Diagnosis costs. The tests used to diagnose MSI-H/dMMR CRC included physical examination, laboratory testing, biomarker testing, colonoscopy, computed tomography scanning, magnetic resonance imaging, and genomic testing (9). Diagnostic costs were estimated using the cost of each test and the proportions of patients who underwent each type of test. The costs of each test were also based on the median value of the price of medical services in 10 representative Chinese provinces or cities, while the proportion of patients undergoing each type of diagnostic test was based on expert opinion. Details are presented in **Appendix Table 4**. The average diagnosis cost was found to be $519.26, which was included in the first treatment cycle.

Patient monitoring costs. The monitoring tests were similar to the diagnostic tests, and thus details were obtained from the same source. The frequency of monitoring tests was obtained from guidelines of CSCO (9) and verified by clinical experts. Notably, the frequency of regorafenib patient monitoring was greater than that of serplulimab patient monitoring because patients receiving regorafenib generally progress faster. Specifically, during the first three years of the PFS state, it was recommended that patients receiving serplulimab and regorafenib were monitored every three months and every two months, respectively. In the fourth and fifth years of the PFS state, it was recommended that both groups of patients were monitored every six months. Patients whose disease did not progress after five years were monitored every 12 months. In the PD state, the frequency of monitoring was identical to that in the first three years of the PFS state (9). Detailed information on patient monitoring is presented in **Appendix Table 5**.

AE management costs. Only grade 3 or above treatment-related AEs that occurred in ≥1% of patients were included in the analysis, as shown in **Table 1**. AEs were classified into two types based on their clinical characteristics: one-off AEs, which only occurred once, and periodic AEs, which could occur in each cycle. Most serplulimab and regorafenib AEs were one-off events, except for hypertension, based on expert opinion. In this study, one-off AEs were included in the first cycle, while periodic AEs were included in each cycle. To calculate the cost of periodic AE management, incidences of periodic AEs that were reported in the trial were converted into rates and respective probabilities. Notably, the AE management costs (see **Table 1**) differed for patients receiving either serplulimab or regorafenib, even for the same AE, based on expert opinion, because serplulimab and regorafenib differ significantly in terms of their pharmacological mechanism. Combining the median values of price of drugs (25) used in the AEs management, the costs for AEs management were estimated.

Hospitalization costs. Hospitalization costs were calculated based on the length of the hospital stay and daily hospitalization costs. On average, patients receiving serplulimab were hospitalized for 1 day in the PFS state and 1.5 days in the PD state, whereas patients receiving regorafenib generally did not require hospitalization in the PFS state, but were hospitalized for 2 days in the PD state in accordance with expert opinion. Daily hospitalization costs were $73.78, based on the median price of medical services of 10 representative Chinese provinces or cities.

Subsequent treatment costs. On the basis of the subsequent treatment data from the ASTRUM-010 trial, we divided subsequent treatment into six categories: chemotherapy, chemotherapy plus targeted therapy, targeted therapy, ICI, radiotherapy, and participation in clinical trials (9). Furthermore, based on expert opinion, in an effort to try more treatment options, most patients (40%) receiving serplulimab accepted targeted therapy after progression, whereas most patients (75%) receiving regorafenib were likely to accept an ICI in clinical practice (see **Appendix Table 6**). The cost of each treatment type was calculated based on the monthly cost and proportion of patients undergoing each treatment type, as shown in **Appendix Table 7**. The costs of subsequent treatment were calculated based on the monthly cost of and proportion of patients undergoing each treatment type. The unit costs were derived from MENET database (25).

End-of-life care costs. End-of-life care costs relate to the cost of treatment prior to death, and were obtained from previous studies (32).

#### 2.2.3 Health state utilities

In the ASTRUM-010 trial, the EuroQoL 5 Dimensions 3 Levels questionnaire was used to measure health utilities. Questionnaires were administered to available patients every four weeks until the end of treatment. The average patient utility value was 0.94 in the PFS state and 0.87 in the PD state.

Because the utilities derived from the ASTRUM-010 trial were higher than those reported by patients who had progressed following standard therapy (17), which might have been inconsistent with the clinical situation, a systematic review was carried out in an effort to obtain more appropriate utility values. It was found that there were no published utility values for MSI-H/dMMR CRC that had either been previously treated or had been metastatic. Consequently, we used utility values derived from Chinese patients with advanced metastatic CRC and assumed that these utility values depended only on their health state, and not on their type of treatment. Thus, the assigned patient utility values were 0.84 in the PFS state and 0.57 in the PD (14), which were used in the base-case analysis.

Disutility values associated with AEs were obtained from previous studies (see **Table 1**). The disutility values for AEs, including the hyperbilirubinemia, impaired liver function, alanine aminotransferase elevated, aspartate aminotransferase elevated and creatine kinase elevated were assumed to be 0, since these AEs were about changes in biochemical indexes and had little impact on patients’ health-related quality of life.

### 2.3 Uncertainty analysis

#### 2.3.1 One-way and probabilistic sensitivity analysis

One-way sensitivity analysis (OWSA) and probabilistic sensitivity analysis (PSA) were undertaken to test for robustness. OWSA was performed with each key parameter varied individually, including a series of costs (e.g., drug acquisition costs, drug administration costs, diagnosis costs, monitoring costs, and AE management costs), the incidences of AEs, utilities, disutilities, and discount rates. If reported in the literature, 95% CIs for base values were used. If the parameter uncertainty was unavailable, a 20% standard variation was used in the OWSA (see **Appendix Table 8**). A tornado diagram of the 10 parameters with the greatest impact on the base-case model results was used to present the OWSA results.

In addition, PSA was conducted to assess the stochastic parametric uncertainty, which provides an estimate of the joint uncertainty of costs and effectiveness, by assigning probabilistic distributions to key input parameters and resampling new values for each parameter from their respective distribution. Specifically, a beta distribution was assigned to incidences of AEs, utilities and disutilities, a gamma distribution was assigned to cost parameters, and probabilistic values of survival parameters were generated using a Cholesky decomposition matrix (see **Appendix Table 8**). This process was repeated using 1000 Monte Carlo simulations.

#### 2.3.2 Scenario analysis

Beyond the base-case analysis, several scenarios were explored to investigate areas of particular uncertainty. For scenario 1, to do the economic evaluation without considering MAIC, direct comparison was performed and the baseline characteristics of patients receiving serplulimab were not adjusted. In scenario 2, the time horizon was limited to 36 months, identical to the follow-up time in the ASTRUM-010 trial, to reduce the uncertainty related to our long-term extrapolation. Because the death risk was assumed twice as that of general population in the base-case analysis, which was conservative, thus, in scenario 3, we set the risk of death of patients in the PFS state or PD state were fourfold as general population, to test the impact of our assumptions regarding the risk of death. In scenario 4, to examine the uncertainty of utility parameters, we used the utility values from the ASTRUM-010 trial and the CORRECT trial, respectively. In the ASTRUM-010 trial, the patient utility value was 0.94 in the PFS state and 0.87 in the PD state. In the CORRECT trial, the patient utility value decreased to 0.73 in the PFS state and 0.59 in the PD state (26).

## 3 Results

### 3.1 Base-case analysis

The costs and health outcomes of the base-case analysis are summarized in **Table 2**. It can be seen that over a lifetime horizon, the total costs for serplulimab and regorafenib were estimated to be $68,722 and $40,106, respectively. In terms of health outcomes, the model predicted that the life expectancies were 7.43 LYs and 1.03 LYs for patients receiving serplulimab and regorafenib, respectively. Adjusting for quality of life, serplulimab provided patients with 6.00 QALYs, while regorafenib provided them with just 0.69 QALYs. Therefore, the ICER was $5,386/QALY and serplulimab was estimated to be cost-effective at the WTP threshold of triple Chinese GDP per capita in 2021 ($36,036).

**Table 2:** Cost effectiveness results.

	Costs ($)	LYs	QALYs	ICER($/QALY)
Base-case				
Regorafenib	40,106.04	1.03	0.69	5,385.94
Serplulimab	68,722.38	7.43	6.00	
Scenario 1: without MAIC				
Regorafenib	40,106.04	1.03	0.69	6,368.74
Serplulimab	77,064.88	8.22	6.49	
Scenario 2: time horizon of 36 months				
Regorafenib	38,433.62	0.97	0.65	20,613.45
Serplulimab	55,134.48	1.85	1.46	
Scenario 3: 4-fold increase in death risk				
Regorafenib	40,037.52	1.03	0.68	6,037.28
Serplulimab	65,966.84	6.18	4.98	
Scenario 4: utility values from other sources				
Regorafenib^a^	40,106.04	1.03	0.92	4,782.81
Serplulimab^a^	68,722.38	7.43	6.91	
Regorafenib^b^	40,106.04	1.03	0.66	6,167.22
Serplulimab^b^	68,722.38	7.43	5.30	

a: using utilities from the ASTRUM-010 trial. b: using utilities from the CORRECT trial. MAIC, matching -adjusted indirect comparison.

### 3.2 Uncertainty analysis

#### 3.2.1 Sensitivity analysis

**Figure 3:**
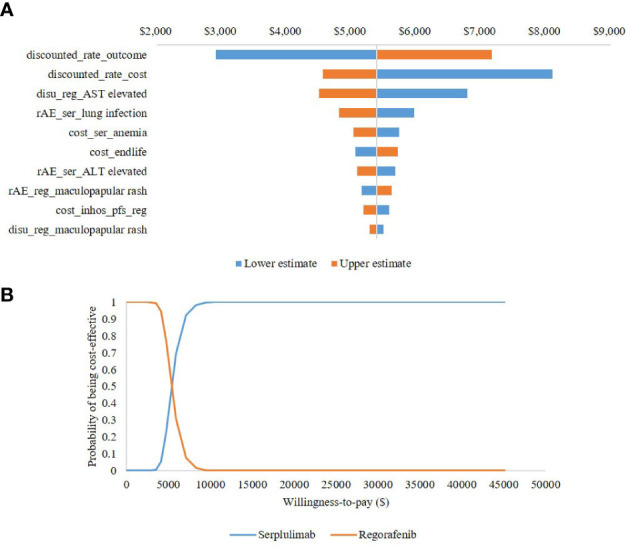
Uncertainty analysis results **(A)** presents tornado diagram of one-way sensitivity analysis. **(B)** presents cost-effectiveness acceptability curve of probabilistic sensitivity analysis. Reg, regorafenib; ser, serplulimab; AE, adverse events; AST, aspartate aminotransferase; ALT, alanine aminotransferase.

It can be seen from the tornado diagram shown in **Figure 3A** that the ICER results were most sensitive to the discount rate of health outcomes, discount rate of costs, and disutility values for elevated aspartate aminotransferase in patients receiving regorafenib. Across all parameters analyzed, the ICER ranged from $2,912 to $8,104 per QALY gained, indicating that serplulimab remained cost-effective. The CEAC shown in **Figure 3B** indicates that at a willingness-to-pay of $36,036 per QALY gained, the probability of serplulimab being the most cost-effective treatment was 100%.

#### 3.2.2 Scenario analysis

The results of the scenario analysis are presented in **Table 2**. In scenario 1, involving direct comparison, incremental QALYs rose to 5.80 at an incremental cost of $369,80.84, and the resulting ICER increased to $6,369/QALY. In scenario 2, serplulimab provided 0.81 more QALYs than regorafenib in the first 36 months, leading to an ICER of $20,613/QALY. In scenario 3, with a fourfold increase in the risk of death, serplulimab provided 4.29 additional QALYs at an incremental cost of $25,929, while the ICER fell to $6,037/QALY. In scenario 4, in which utility values from the ASTRUM-010 trial were used, the ICER was $4,783/QALY, lower than the figure in the base-case analysis. When utility values from the CORRECT trial were used, the ICER was $6,167/QALY. All of the ICERs estimated in the scenario analyses remained under the WTP threshold, confirming that serplulimab was more cost-effective than regorafenib for the treatment of previously treated unresectable or metastatic MSI-H/dMMR CRC.

## 4 Discussion

Although patients with MSI-H/dMMR constitute a limited proportion of CRC patients, it is likely that the number of patients with MSI-H/dMMR CRC will increase with the rising number of cases of CRC, both in China and elsewhere (1, 2, 33, 34). For these patients, clinical studies suggest that they could gain considerable benefit from treatment with ICIs because MSI-H/dMMR tumors are highly infiltrated by immune cells, which show dramatic responses to several ICIs (35). Nevertheless, none of these ICIs has been included in China’s NRDL, resulting in unmet clinical needs and a significant financial burden for patients. Hence, a cost-effectiveness analysis of ICIs used for the treatment of MSI-H/dMMR CRC is necessary to assist reimbursement decision-making. Given the available ICIs for the treatment of MSI-H/dMMR CRC in China and the government’s policy regarding the dynamic adjustment of the NRDL, we selected regorafenib from the NRDL as a comparator and evaluated the cost-effectiveness of serplulimab, providing evidence for the reimbursement decision-making of the NHSA and improving the allocation efficiency of medical resources in China.

### 4.1 Main findings

Our findings showed that serplulimab was more cost-effective than regorafenib for treatment of MSI-H/dMMR CRC, with an ICER significantly under the WTP threshold. One reason was that patients with MSI-H/dMMR CRC were sensitive to ICIs (35), and the treatment with serplulimab provided significantly improved health outcomes (6.00 QALYs). Another reason was that despite treatment with serplulimab incurring higher drug acquisition costs, it was associated with lower costs for AE management, subsequent treatment, and end-of-life care, resulting in relatively small incremental total costs. The robustness of the base-case results was confirmed by OWSA and PSA. Our scenario analysis also confirmed that the results were robust to variations in key parameters and assumptions. Scenario 1 showed a higher ICER ($6,369/QALY) than that in the base-case because of better baseline characteristics of patients in the ASTRUM-010 trial, which resulted in higher costs for patients receiving serplulimab because of their longer survival. Scenario 2 involving a limited time horizon (36 months) increased the ICER ($20,613/QALY) significantly because the health outcomes of serplulimab could not be fully captured within the follow-up period, whereas most of the costs were occurred in the first three years. In scenario 3, with a fourfold increase in the risk of death, patients in the PFS or PD states proceeded to death more quickly, and thus the reduction in health outcomes was greater than the increase in total costs, increasing the ICER. Scenario 4 resulted in the same total costs and LYs as the base-case analysis, and thus the ICER declined when the higher utility values from the ASTRUM-010 trial were used and rose when the lower utility values from the CORRECT trial were used.

To the best of our knowledge, there have been four modelling studies also investigating the cost-effectiveness of PD-1 inhibitors for the treatment of MSI-H/dMMR CRC (36–39). These four studies were conducted in the US and China context, in general, PD-1 inhibitors were cost-effective, which were consistent with our findings. Specifically, pembrolizumab was cost-effective both in first- and second-line due to superiority of efficacy compared with chemotherapy (37–39). In addition, in the study comparing ipilimumab plus nivolumab, nivolumab alone and chemotherapy, ICIs were not cost-effective in the base-case analysis due to their high price. However, when the treatment duration was limited to two years, ICIs were still cost-effective with an ICER under the WTP threshold of $100,000 in the US (36).

### 4.2 Strengths and limitations

This is the first cost-effectiveness analysis of serplulimab compared with regorafenib in patients with previously treated unresectable or metastatic MSI-H/dMMR CRC, and thus this study has several strengths in terms of research methods and data acquisition. First, most of the patients receiving serplulimab or regorafenib treatment were Chinese, providing contextual relevance for Chinese decision-making. Second, MAIC was conducted to improve the comparability of the efficacy of the two treatment regimens. An analysis was also conducted without MAIC in an effort to identify the impact of MAIC on the results, confirming that it favored regorafenib. Third, a mixture cure model and increased risk of death were included to address the plateau problem in relation to the survival curve with the aim of providing more accurate estimates of lifelong health outcomes and more informed economic evaluations. Fourth, experts were invited to share their opinions on comparator selection, diagnosis, monitoring, AEs, and subsequent treatment, more closely aligning the study with medical care in practice.

This study also has some limitations. First, the comparator was not one of the abovementioned ICIs. This was because clinical trials of pembrolizumab and envafolimab were set up for first-line treatment (10, 11) and untreated patients in those trials were expected to have significantly better outcomes than patients treated with serplulimab, and thus we believed that they were not suitable for comparison with patients treated with serplulimab. As for tislelizumab, the published data were focused on treatment of MSI-H/dMMR solid tumors, of which CRC only accounted for 61.3% and the median PFS was not reached (12). Conversely, it is necessary to compare innovative treatments with those already in the NRDL if they are to be considered for inclusion in the NRDL. Therefore, regular treatment with regorafenib, which was on the NRDL and met the requirements of the NHSA, when lacking ICIs for MSI-H/dMMR CRC was selected as the comparator. Second, MSI-H/dMMR CRC is a subtype of CRC with small numbers of patients, and thus the MSI/MMR status was actually unknown in the CONCUR trial, and therefore the target population of interest in relation to regorafenib was likely to be substantially underrepresented (4, 14). Third, the increased risk of death and other parameters that were derived based on expert opinion might involve uncertainty as a result of the inevitable subjectivity and sample bias. However, the results of the sensitivity analysis and scenario analysis showed that the ICER only changed moderately and remained well under the WTP threshold. Fourth, the survival data in relation to serplulimab treatment was immature, and thus long-term extrapolation might include uncertainty. Fifth, the cost and disutility parameters from expert opinions and assumptions might be uncertain, but according to the OWSA, they may not have significant impact on model results. Future studies might provide more information by choosing ICIs as comparators, using clinical trials with extended follow-up times, or incorporating real-world evidence.

## 5 Conclusion

Despite the various abovementioned uncertainties, the results of our study, which was based on unanchored MAIC, suggest that serplulimab has significant efficacy over a lifetime horizon and is more cost-effective than regorafenib for the treatment of previously treated unresectable or metastatic MSI-H/dMMR CRC in China.

## Data availability statement

The data analyzed in this study is subject to the following licenses/restrictions: The individual patient data from ASTRUM-010 trial is not available to the public. Requests to access these datasets should be directed to Yue Ma, cpumayue@163.com.

## Author contributions

AM and HL takes responsibility for the data source and the accuracy of the modeling analysis. Study design, YM and HL. Literature search, data extraction, and expert interview, JZ, YY, and XW. Drafting of manuscript, YM and JZ. Critical revision of the manuscript, AM and HL. All authors contributed to the article and approved the submitted version.
