# Supplementary material for: The cost-effectiveness analysis of serplulimab versus regorafenib for treating previously treated unresectable or metastatic microsatellite instability-high or deficient mismatch repair colorectal cancer in China
Source: Front Oncol. 2023 Apr 26;13:1113346. doi: 10.3389/fonc.2023.1113346 (PMC10171919; doi:10.3389/fonc.2023.1113346)
Supplement: Supplementary file 1 [file DataSheet_1.docx]

**Appendix**

**Appendix Table 1. Baseline characteristics of the ASTRUM-101 and CONCUR trial**

|  | Original characteristics | | MAIC | |
| --- | --- | --- | --- | --- |
|  | Serplulimab  (n=74) | Regorafenib  (n=136) | Serplulimab  (ESS=48.6) | Regorafenib  (n=136) |
| Age (years) |  |  |  |  |
| ＜65 | 86% | 70% | 70% | 70% |
| ≥65 | 14% | 30% | 30% | 30% |
| Sex |  |  |  |  |
| Men | 58% | 63% | 63% | 63% |
| Female | 42% | 38% | 38% | 38% |
| ECOG performance status |  |  |  |  |
| 0 | 47% | 26% | 26% | 26% |
| 1 | 53% | 74% | 74% | 74% |
| Histology |  |  |  |  |
| Adenocarcinoma | 96% | 96% | 96% | 96% |
| Mucinous carcinoma | 4% | 4% | 4% | 4% |
| Number of metastatic sites |  |  |  |  |
| Single | 26% | 21% | 21% | 21% |
| Multiple | 74% | 79% | 79% | 79% |
| Previous systemic anticancer treatment lines | | | | |
| 1-2 | 28% | 35% | 35% | 35% |
| ≥3 | 72% | 62% | 62% | 62% |
| Previous targeted biological treatment | | | | |
| None | 35% | 41% | 41% | 41% |
| Any | 65% | 59% | 59% | 59% |

ECOG: Eastern Cooperative Oncology Group


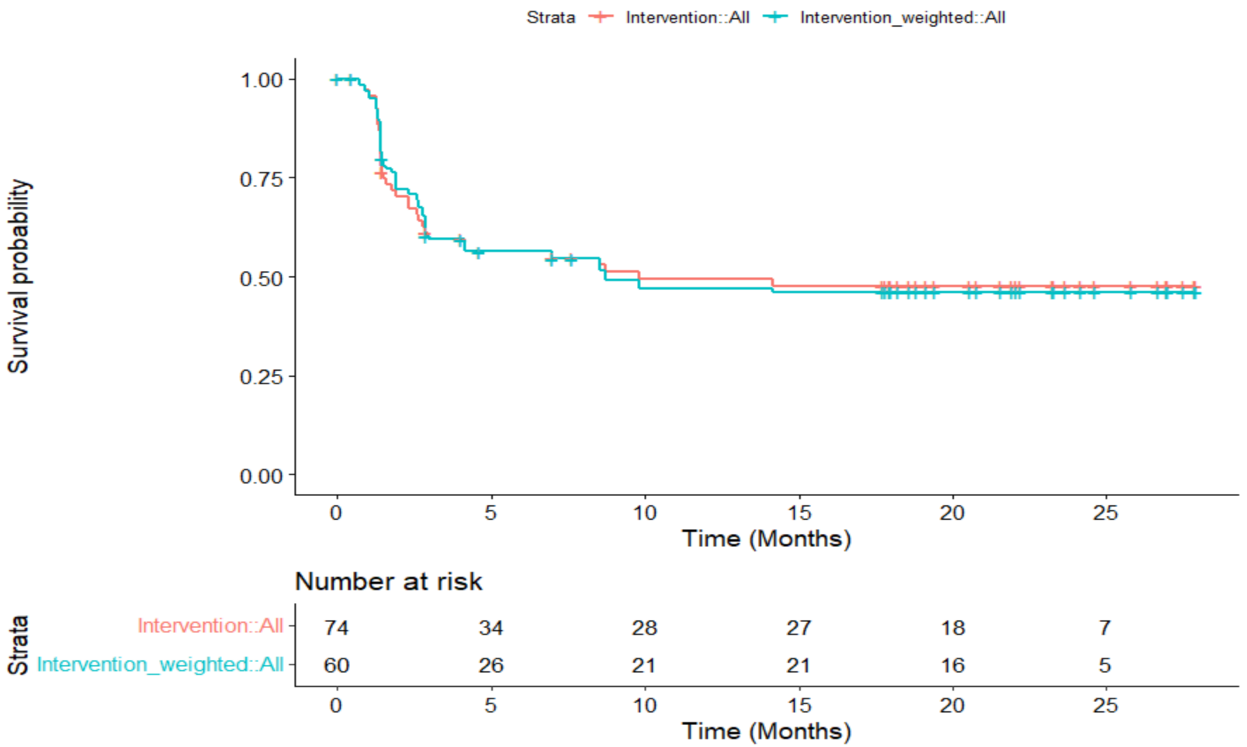
**a**

**
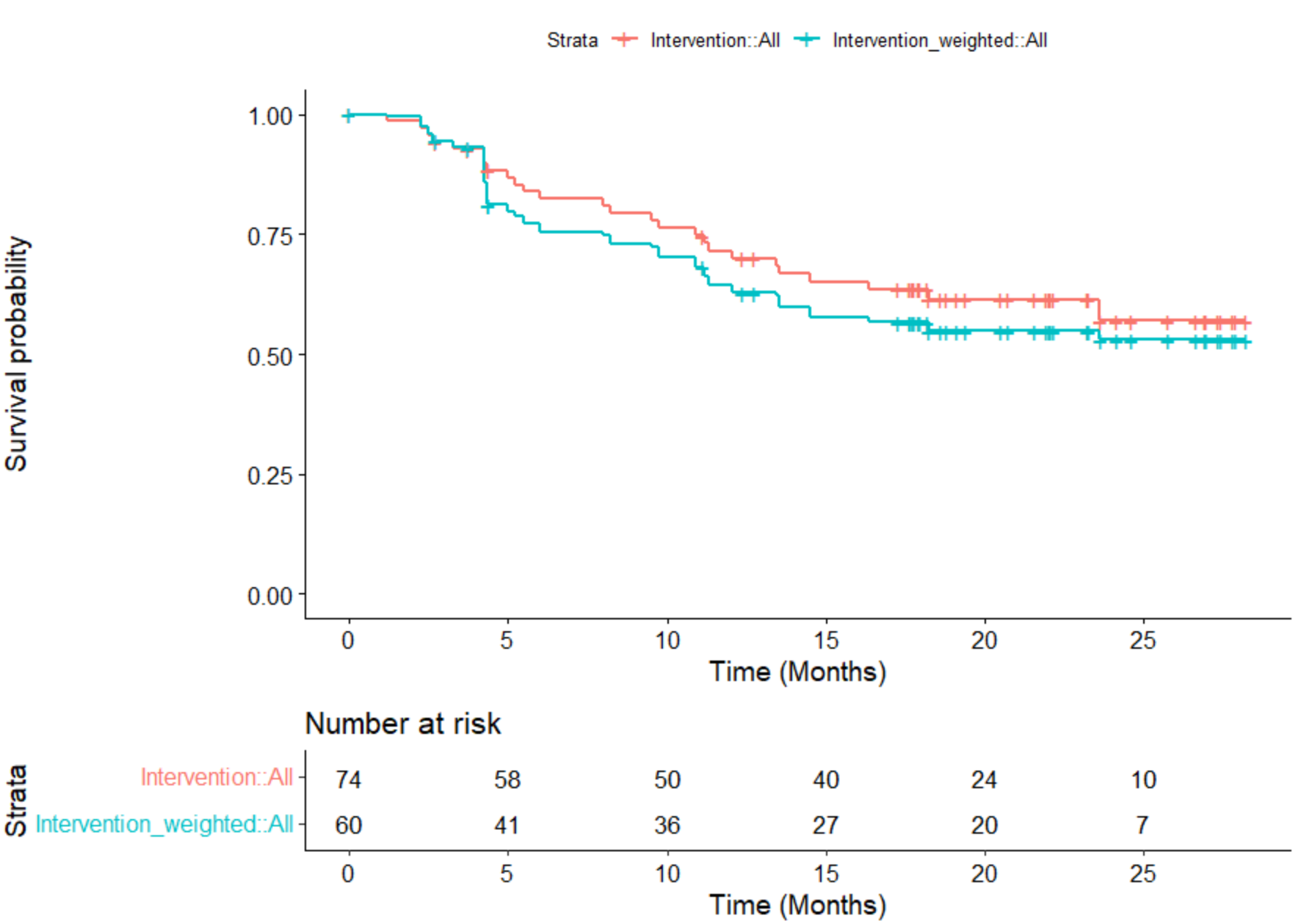
b**

（a）presents original and weighted progression free survival curves of serplulimab. (b) presents original and weighted overall survival curves of serplulimab.

**Appendix Figure 1. Survival curves using matching-adjusted indirect comparison in serplulimab.**

**Appendix Table 2. AIC and BIC of 6 parametric distributions in serplulimab**

|  | **AIC** | **BIC** |
| --- | --- | --- |
| **PFS (standard model)** | | |
| Exponential | 221.25 | 223.55 |
| Gamma | 214.03 | 218.64 |
| **Gompertz** | **191.05** | **195.66** |
| Weibull | 211.24 | 215.85 |
| Log-Logistic | 205.70 | 210.30 |
| Log-Normal | 202.36 | 206.97 |
| **OS (mixture cure model)** | | |
| Exponential | 212.70 | 217.31 |
| Gamma | 207.83 | 214.74 |
| Gompertz | 212.22 | 219.13 |
| Weibull | 209.03 | 215.95 |
| Log-Logistic | 207.59 | 214.50 |
| **Log-Normal** | **206.43** | **213.35** |

AIC: Akaike information criterion, BIC: Bayesian information criterion, PFS: Progression free survival, OS: overall survival

**Appendix Table 3. AIC and BIC of 6 parametric distributions in regorafenib**

|  | **AIC** | **BIC** |
| --- | --- | --- |
| **PFS (standard model)** | | |
| Exponential | 605.32 | 608.23 |
| Gamma | 592.10 | 597.92 |
| Gompertz | 606.30 | 612.13 |
| Weibull | 597.87 | 603.69 |
| **Log-Logistic** | **580.52** | **586.35** |
| Log-Normal | 582.99 | 588.81 |
| **OS (standard model)** | | |
| Exponential | 743.71 | 746.62 |
| Gamma | 716.16 | 721.98 |
| Gompertz | 724.37 | 730.19 |
| Weibull | 716.88 | 722.71 |
| **Log-Logistic** | **723.86** | **683.31** |
| Log-Normal | 725.03 | 730.85 |

AIC: Akaike information criterion, BIC: Bayesian information criterion, PFS: Progression free survival, OS: overall survival

**Appendix Table 4. Diagnostic tests and costs**

| **Diagnostic test** | **Proportion of patients** | **Costs ($)** | **Source** |
| --- | --- | --- | --- |
| Physical examination | 100% | 4.55 | The proportion was derived from expert opinion and the cost was taken from 10 representative provinces or cities in China. |
| Laboratory testing |  |  |  |
| Blood routine | 100% | 2.32 |  |
| Urine routine | 100% | 1.06 |  |
| Stool routine | 100% | 0.85 |  |
| Fecal occult blood test | 100% | 0.89 |  |
| Biochemistry test | 100% | 42.43 |  |
| Biomarker test (CEA, AFP, CA199 and CA125) | 100% | 29.19 |  |
| Colonoscopy | 10% | 26.28 |  |
| Contrast CT scan (chest, abdomen and pelvis) | 5% | 149.43 |  |
| Contrast and non-contrast CT scan (abdomen and pelvis) | 5% | 179.69 |  |
| CT scan (chest) | 90% | 30.11 |  |
| PET-CT | 10% | 812.99 |  |
| MRI (pelvis) | 10% | 70.70 |  |
| Contrast MRI (liver) | 45% | 79.36 |  |
| Genetic test | 100% | 267.70 |  |

CEA: carcinoembryonic antigen, AFP: alpha fetoprotein, CT: computed tomography, CA 199: carbohydrate antigen 199, CA125: carbohydrate antigen 125, PET: positron emission tomography, MRI: magnetic resonance imaging

**Appendix Table 5. Patient monitoring information of patients treated by serplulimab and regorafenib**

|  | **Proportion of patients^a^** | **Frequency of visit per year^a^** | | | | | | | | **Costs^b^**  **($)** |
| --- | --- | --- | --- | --- | --- | --- | --- | --- | --- | --- |
|  |  | **the first 3 years in PFS** | | **4^th^-5^th^ year in PFS** | | **After 5^th^ year in PFS** | | **PD** | |  |
|  |  | Serplulimab | Regorafenib | Serplulimab | Regorafenib | Serplulimab | Regorafenib | Serplulimab | Regorafenib |  |
| Physical examination | 100% | 4 | 6 | 2 | 2 | 1 | 1 | 4 | 6 | 4.55 |
| Laboratory testing | | | | | | | | | | |
| Blood routine | 100% | 4 | 6 | 2 | 2 | 1 | 1 | 4 | 6 | 2.32 |
| Urine routine | 100% | 4 | 6 | 2 | 2 | 1 | 1 | 4 | 6 | 1.06 |
| Liver and kidney function | 100% | 4 | 6 | 2 | 2 | 1 | 1 | 4 | 6 | 24.40 |
| Biomarker test (CEA and CA199) | 100% | 4 | 6 | 2 | 2 | 1 | 1 | 4 | 6 | 17.39 |
| CT scan (Chest, abdomen and pelvis) | 100% | 4 | 6 | 2 | 2 | 1 | 1 | 4 | 6 | 120.45 |
| MRI (Chest, abdomen and pelvis) | 40% | 4 | 6 | 2 | 2 | 1 | 1 | 4 | 6 | 70.70 |
| PET-CT | 5.50% | 4 | 6 | 2 | 2 | 1 | 1 | 4 | 6 | 812.99 |

^a^ Frequency of follow-up visit and the proportion of patients in each test was from expert opinion.

^b^ Cost is the median price of each test in 10 representative provinces and cities.

CEA: carcinoembryonic antigen, CA 199: carbohydrate antigen 199, CT: computed tomography, MRI: magnetic resonance imaging, PET: positron emission tomography, PFS: Progression free survival, OS: overall survival

**Appendix Table 6. Subsequent treatment pattern for patients treated by serplulimab and regorafenib**

| **Treatment pattern** | **Serplulimab** | **Regorafenib** | **Source** |
| --- | --- | --- | --- |
| Chemotherapy | 10% | 0% | Expert opinion |
| Chemotherapy+ targeted therapy | 10% | 0% |  |
| Targeted therapy | 40% | 0% |  |
| Immune checkpoint inhibitors | 0% | 75% |  |
| Radiotherapy | 15% | 0% |  |
| Clinical trial | 25% | 25% |  |

**Appendix Table 7. Subsequent treatment options and costs**

| **Subsequent therapy** | **Proportion of patients** | **Drug** | **Administration** | **Dosage** | **Unit cost ($)** | **Usage per unit** | **Costs of each treatment per cycle ($)** | **Cost of each pattern per cycle ($)** |
| --- | --- | --- | --- | --- | --- | --- | --- | --- |
| **Chemotherapy** | | | | | | | | |
| Oxaliplatin | 16.67% | Oxaliplatin | 85mg/m2, iv drip, q2w | 50mg | 123.18 | 6.00 | 739.08 | 1,083.69 |
| Gemcitabine | 16.67% | Gemcitabine | 1000mg/m2, iv drip, once weekly for 3 weeks of each 28-day cycle | 200mg | 19.33 | 24.00 | 463.83 |  |
| Capecitabine | 8.33% | Capecitabine | 1250mg/m2, po, bid, day1-day14 of each 21-day cycle | 500mg | 17.24 | 12.44 | 214.50 |  |
| Vinorelbine Tartrate Capsules | 8.33% | VinorelbineTartrate Capsules | 10-40mg/d, po, qd, day1-day21 of each 28-day cycle | 20mg | 122.27 | 26.00 | 3,179.09 |  |
| Raltitrexed | 25.00% | Raltitrexed | 3mg/m2, iv drip, q3w | 2mg | 99.24 | 6.40 | 635.13 |  |
| Irinotecan | 16.67% | Irinotecan | 180mg/m2, iv drip, q2w | 40mg | 102.16 | 14.40 | 1,471.12 |  |
| Trifluridine-tipiracil | 8.33% | Trifluridine-tipiracil | 35mg/m2, po, qd, day1-5 and day8-12 of each 28-day cycle | 15mg | 631.41 | 3.73 | 2,357.25 |  |
| **Chemotherapy + targeted therapy** | | | | | | | | |
| Chemotherapy + Bevacizumab | 70% | Bevacizumab | 5mg/kg, iv drip, q2w | 100mg | 193.75 | 2.00 | 387.50 |  |
|  |  | Oxaliplatin | 85mg/m2, iv drip, q2w | 50mg | 123.18 | 6.00 | 739.08 | 1,902.98 |
|  |  | Calcium Levofolinate Hydrate | 400mg/m2, iv drip, q2w | 25mg | 7.03 | 52.00 | 365.62 |  |
|  |  | 5-Fluorouracil | 400mg/m2, iv, on day 1; 2400mg, iv drip on day 2, q2w | 250mg | 11.66 | 36.00 | 419.84 |  |
| Chemotherapy + Cetuximab | 30% | Cetuximab | 500mg/m2, iv, q2w | 100mg | 178.65 | 2.00 | 357.30 |  |
|  |  | Oxaliplatin | 85mg/m2, iv, q2w | 50mg | 123.18 | 6.00 | 739.08 |  |
|  |  | Calcium Levofolinate Hydrate | 400mg/m2, iv drip, q2w | 25mg | 7.03 | 52.00 | 365.62 |  |
|  |  | 5-Fluorouracil | 400mg/m2, iv, on day 1; 2400mg, iv drip on day 2, q2w | 250mg | 11.66 | 36.00 | 419.84 |  |
| **Targeted therapy** | | | | | | | | |
| Denosumab | 14.29% | Denosumab | 120mg, ih, q4w | 60mg | 243.99 | 1.00 | 243.99 | 1,083.92 |
| Regorafenib | 14.29% | Regorafenib | 160mg po, once daily on day1-21 of 28-day cycle | 40mg | 716.40 | 3.00 | 2,149.19 |  |
| Fruquintinib | 57.14% | Fruquintinib | 5mg, po, once daily on day1-21 of 28-day cycle | 5mg | 392.51 | 3.00 | 1,177.52 |  |
| Apatinib | 14.29% | Apatinib | 850mg, po, qd | 250mg | 161.41 | 3.00 | 484.22 |  |
| **Immune checkpoint inhibitors** | | | | | | | | |
| Camrelizumab | 28.57% | Camrelizumab | 3mg/kg, iv drip, q3w | 200mg | 434.34 | 1.33 | 579.12 | 2,399.62 |
| Pembrolizumab | 28.57% | Pembrolizumab | 200mg, iv drip, q3w | 100mg | 2,657.94 | 2.67 | 7087.85 |  |
| Toripalimab | 42.86% | Toripalimab | 3mg/kg, iv drip, q2w | 80mg | 121.95 | 4.00 | 487.80 |  |
| Tislelizumab | 0.00% | Tislelizumab | 200mg, iv drip, q3w | 100mg | 323.38 | 1.33 | 431.17 |  |
| Envafolimab | 0.00% | Envafolimab | 200mg, ih, q4w | 200mg | 887.07 | 1.00 | 887.07 |  |
| **Radiotherapy** | 100% | - | - | - | 116.46 | 1.00 | 116.46 | 116.46 |
| **Clinical trial** | 100% | - | - | - | 0.00 | 0.00 | 0.00 | 0.00 |

iv: intravenous infusion, po: by mouth, ih: hypodermic injection

q2w: every 2 weeks, q3w: every 3 weeks, q4w: every 4 weeks, qd: once daily, bid: twice a day

**Appendix Table 8. Model inputs**

|  | **Serplulimab** | | | **Regorafenib** | | |  |  |
| --- | --- | --- | --- | --- | --- | --- | --- | --- |
| **Parameter** | **Base-case value** | **Range** | | **Base-case value** | **Range** | | **Distribution** | **Reference** |
|  |  | **Minimum** | **Maximum** |  | **Minimum** | **Maximum** |  |  |
| **Cost ($)** |  |  |  |  |  |  |  |  |
| Diagnosis | 519.26 | 415.41 | 623.11 | 519.26 | 415.41 | 623.11 | Gamma | Estimation* |
| Drug acquisition costs per cycle | 3,315.68 |  |  | 2,149.19 |  |  | Gamma |  |
| Follow-up costs per cycle |  |  |  |  |  |  |  | Estimation* |
| The first 3 years in PFS state | 75.65 | 60.52 | 90.78 | 113.48 | 90.78 | 136.17 | Gamma |  |
| The 4^th^-5^th^ year in PFS state | 37.83 | 30.26 | 45.39 | 37.83 | 30.26 | 45.39 | Gamma |  |
| After 5^th^ year in PFS state | 18.91 | 15.13 | 22.70 | 18.91 | 15.13 | 22.70 | Gamma |  |
| In PD state | 75.65 | 60.52 | 90.78 | 113.48 | 90.78 | 136.17 | Gamma |  |
| AE management costs |  |  |  |  |  |  |  | Estimation* |
| Anemia | 6.96 | 5.57 | 8.35 | - |  |  | Gamma |  |
| Hyperbilirubinemia | 76.16 | 60.92 | 91.39 | 37.92 | 30.33 | 45.50 | Gamma |  |
| Impaired liver function | 76.16 | 60.92 | 91.39 | - |  |  | Gamma |  |
| Alanine aminotransferase elevated | 76.16 | 60.92 | 91.39 | 37.92 | 30.33 | 45.50 | Gamma |  |
| Aspartate aminotransferase elevated | - |  |  | 37.92 | 30.33 | 45.50 | Gamma |  |
| Lung infection | 88.85 | 71.08 | 106.62 | - |  |  | Gamma |  |
| Neutropenia | 62.62 | 50.10 | 75.15 | 84.93 | 67.94 | 101.91 | Gamma |  |
| Leukopenia | 62.62 | 50.10 | 75.15 | 26.48 | 21.18 | 31.77 | Gamma |  |
| Thrombocytopenia | - |  |  | 2,139.77 | 1,711.81 | 2,567.72 | Gamma |  |
| Diarrhea | 2.95 | 2.36 | 3.54 | 12.04 | 9.63 | 14.45 | Gamma |  |
| Creatine kinase elevated | 3.28 | 2.62 | 3.93 | - |  |  | Gamma |  |
| Hypertension | 0.18 | 0.14 | 0.21 | 0.18 | 0.14 | 0.21 | Gamma |  |
| Hand foot skin reaction | - |  |  | 16.18 | 12.94 | 19.42 | Gamma |  |
| Maculopapular rash | - | - | - | 17.63 | 14.11 | 21.16 | Gamma |  |
| Subsequent treatment costs per cycle | 749.71 | 599.76 | 899.65 | 1,799.71 | 1,439.77 | 2,159.66 | Gamma | Estimation* |
| Hospitalization costs |  |  |  |  |  |  |  | Estimation* |
| In PFS state | 73.38 | 58.71 | 88.06 | 0.00 | 0.00 | 0.00 | Gamma |  |
| In PD state | 110.08 | 88.06 | 132.09 | 146.77 | 117.41 | 176.12 | Gamma |  |
| Administration costs per cycle |  |  |  |  |  |  |  | Estimation* |
| In PFS state | 2.97 | 2.37 | 3.56 | 0.00 | 0.00 | 0.00 | Gamma |  |
| In PD state | 1.67 | 1.34 | 2.00 | 1.80 | 1.44 | 2.16 | Gamma |  |
| End-of-life care costs | 2,046.84 | 1,637.47 | 2,456.21 | 2,046.84 | 1,637.47 | 2,456.21 | Gamma | [1] |
| **Utility values** |  |  |  |  |  |  |  |  |
| PFS | 0.84 | 0.672 | 1 | 0.84 | 0.672 | 1 | Beta | [2] |
| PD | 0.57 | 0.456 | 0.684 | 0.57 | 0.456 | 0.684 | Beta | [2] |
| **Disutility values** |  |  |  |  |  |  |  |  |
| Anemia | 0.085 | 0.068 | 0.102 | - |  |  | Beta | [3] |
| Hyperbilirubinemia | 0 | 0 | 0 | 0 | 0 | 0 | Beta | / |
| Impaired liver function | 0 | 0 | 0 | - |  |  | Beta | / |
| Alanine aminotransferase elevated | 0 | 0 | 0 | 0 | 0 | 0 | Beta | / |
| Aspartate aminotransferase elevated | - |  |  | 0 | 0 | 0 | Beta | / |
| Lung infection | 0.195 | 0.156 | 0.234 | - |  |  | Beta | [4] |
| Neutropenia | 0.0607 | 0.04856 | 0.07284 | 0.0607 | 0.04856 | 0.07284 | Beta | [3] |
| Leukopenia | 0.0607 | 0.04856 | 0.07284 | 0.0607 | 0.04856 | 0.07284 | Beta | [3] |
| Thrombocytopenia | - |  |  | 0.19 | 0.152 | 0.228 | Beta | [5] |
| Diarrhea | 0.07 | 0.056 | 0.084 | 0.07 | 0.056 | 0.084 | Beta | [5] |
| Creatine kinase elevated | 0 | 0 | 0 | - |  |  | Beta | / |
| Hypertension | 0.04 | 0.032 | 0.048 | 0.04 | 0.032 | 0.048 | Beta | [5] |
| Hand foot skin reaction | - |  |  | 0.116 | 0.0928 | 0.1392 | Beta | [6] |
| Maculopapular rash | - |  |  | 0.03248 | 0.025984 | 0.038976 | Beta | [3] |
| **Incidence of AEs (grade 3/4)** |  |  |  |  |  |  |  | ASTRUM-010 and [2] |
| Anemia | 10.81% | 8.65% | 12.97% | - |  |  | Beta |  |
| Hyperbilirubinemia | 6.76% | 5.41% | 8.11% | 6.00% | 4.80% | 7.20% | Beta |  |
| Impaired liver function | 5.41% | 4.33% | 6.49% | - |  |  | Beta |  |
| Alanine aminotransferase elevated | 1.35% | 1.08% | 1.62% | 7.00% | 5.60% | 8.40% | Beta |  |
| Aspartate aminotransferase elevated | - |  |  | 6.00% | 4.80% | 7.20% | Beta |  |
| Lung infection | 2.70% | 2.16% | 3.24% | - |  |  | Beta |  |
| Neutropenia | 4.05% | 3.24% | 4.86% | 2.00% | 1.60% | 2.40% | Beta |  |
| Leukopenia | 2.70% | 2.16% | 3.24% | 2.00% | 1.60% | 2.40% | Beta |  |
| Thrombocytopenia | - |  |  | 3.00% | 2.40% | 3.60% | Beta |  |
| Diarrhea | 2.70% | 2.16% | 3.24% | 1.00% | 0.80% | 1.20% | Beta |  |
| Creatine kinase elevated | 2.70% | 2.16% | 3.24% | - |  |  | Beta |  |
| Hypertension | 2.70% | 2.16% | 3.24% | 11.00% | 8.80% | 13.20% | Beta |  |
| Hand foot skin reaction | - |  |  | 16.00% | 12.80% | 19.20% | Beta |  |
| Maculopapular rash | - |  |  | 4.00% | 3.20% | 4.80% | Beta |  |

*The estimated values were calculated based on expert opinion and prices form 10 representative provinces or cities in China. PFS: Progression free survival, PD: Progression disease, AE: adverse event

Reference

1. Chiang JK, Kao YH. The impact of hospice care on survival and cost saving among patients with liver cancer: a national longitudinal population-based study in Taiwan. Support Care Cancer. 2015 Apr;23(4):1049-55.
2. Li J, Qin S, Xu R, Yau TC, Ma B, Pan H, et al. Regorafenib plus best supportive care versus placebo plus best supportive care in Asian patients with previously treated metastatic colorectal cancer (CONCUR): a randomised, double-blind, placebo-controlled, phase 3 trial. Lancet Oncol. 2015 Jun;16(6):619-29.
3. Tikhonova IA, Huxley N, Snowsill T, Crathorne L, Varley-Campbell J, Napier M, et al. Economic Analysis of First-Line Treatment with Cetuximab or Panitumumab for RAS Wild-Type Metastatic Colorectal Cancer in England. Pharmacoeconomics. 2018 Jul;36(7):837-851.
4. Barbier M, Durno N, Bennison C, Örtli M, Knapp C, Schwenkglenks M. Cost-effectiveness and budget impact of venetoclax in combination with rituximab in relapsed/refractory chronic lymphocytic leukemia in Switzerland. Eur J Health Econ. 2022 Jul;23(5):837-846.
5. Nafees B, Lloyd AJ, Dewilde S, Rajan N, Lorenzo M. Health state utilities in non-small cell lung cancer: An international study. Asia Pac J Clin Oncol. 2017 Oct;13(5):e195-e203.
6. Lloyd A, Nafees B, Narewska J, Dewilde S, Watkins J. Health state utilities for metastatic breast cancer. Br J Cancer. 2006 Sep 18;95(6):683-90.
